# A novel compound heterozygous mutation in the arginase-1 gene identified in a Chinese patient with argininemia: A case report

**DOI:** 10.1097/MD.0000000000021634

**Published:** 2020-08-07

**Authors:** Dongqing Cui, Yanxia Liu, Liang Jin, Liping Hu, Lili Cao

**Affiliations:** Department of Neurology, Qilu Hospital of Shandong University, Jinan, Shandong, People's Republic of China.

**Keywords:** *ARG1* gene, argininemia, progressive spastic diplegia

## Abstract

**Introduction:**

Arginineemia, also known as arginase deficiency, is a rare autosomal recessive metabolic disease. The diagnosis sometimes may be delayed due to atypical clinical manifestations. Confirmation of arginineemia depends on genetic testing.

**Patient concerns:**

We reported a Chinese male child presenting with hyperargininemia and progressive spastic diplegia, who has a novel compound heterozygous mutation in the arginase-1 (*ARG1*) gene (c.263-266delAGAA, p.K88Rfs^∗^45;c.674T>C,p.L216P), respectively, coming from his mother and father.

**Diagnosis:**

The patient was diagnosed with argininemia with a novel compound homozygous mutation of the *ARG1* gene at the age of 12 years.

**Interventions:**

The patient had a low-protein diet (0.8 g/kg/day). Baclofen, eperisone hydrochloride, botulinum toxin, and rehabilitation training were used to improve his spastic diplegia symptoms for 3 months.

**Outcomes:**

The patient's blood arginine was still high after 3 months’ low-protein diet. His spastic diplegia symptoms had not aggravated after 3 months’ treatment.

**Conclusions:**

Argininemia should be considered in a patient with slowly progressive neurologic manifestations, especially spastic diplegia. This case also suggests that tandem mass spectrometry should be used as an effective tool in the validity of neonatal screening for early diagnosis.

## 1 Introduction

Argininemia, caused by a deficiency of arginase 1 whose role is to decompose of arginine into ornithine and urea, is a rare autosomal recessive urea cycle disorder and according to the data from newborn screening records and the urea cycle disorders (UCDs) consortium's study, the incidence of arginase 1 deficiency has been estimated at about 1:950,000,^[1]^ accounting for 3.5% of all UCDs.^[2]^ Unlike other urea cycle disorders, patients with arginineemia rarely develop hyperammonemia. Rather, some nervous system dysfunctions including elastic paraplegia, seizures, cognitive dysfunction, ataxia, stunting, and mental retardation, are usually presented in patients with argininemia. Currently, the understanding of this disease remains in the stage of case reports rather than a unified diagnosis and treatment guideline. We described a male child with hyperargininemia and progressive spastic diplegia and reported a novel compound heterozygous mutation in *ARG1* gene (c.263-266delAGAA, p.K88Rfs^∗^45;c.674T>C,p.L216P) for the first time. The patient's symptoms are not aggravated by limiting protein intake, taking medications, and doing rehabilitation training.

## 2 Case Report

The study has been approved by Scientific Research Ethics Committee of Qilu Hospital, Shandong University. The patient and his parent provided informed consent for publication of this case.

The patient, as the first child of a Chinese parent who was not consanguineous, was born in 38 + 5 weeks’ gestation with a birth weight of 3100 g, a head circumference of 32 cm, and a body length of 50 cm. The family and histories of the patient are normal. He has not presented any feeding abnormality of food. He has a younger sister who is developing normally.

The patient presented to Neurosurgery Clinic in our hospital at the age of 12 years and 9 months because he had gradually increasing gait abnormalities for almost 4 years, showing unstable walking, foot toe landing, hip flexion, adduction, internal rotation, foot drop, and varus. At this time, the patient had a height of 150 cm and a weight of 50 kg. Then, he was arranged a brain magnetic resonance examination, which showed a space-occupying lesion in the fourth ventricle, and then the patient was admitted to the hospital for surgical resection. Histological pathology of the removed lesion showed cholesteatoma. Laboratory tests revealed mildly elevated prothrombin time (14.6 seconds, normal range: 8.80–13.80 seconds), prothrombin normalization ratio (1.32, normal range: 0.8–1.2) and d-dimer (2.54 μg/mL, normal range: <0.50 μg/mL) while mildly decreased prothrombin time activity (66%, normal range: 70%—140%) and fibrinogen (1.94 g/L, normal range: 2.00–4.00 g/L), but normal blood homocysteine, hepatic enzyme level, and bilirubin level. However, there was no significant improvement in this boy's gait abnormality after operation.

**Figure 1:**
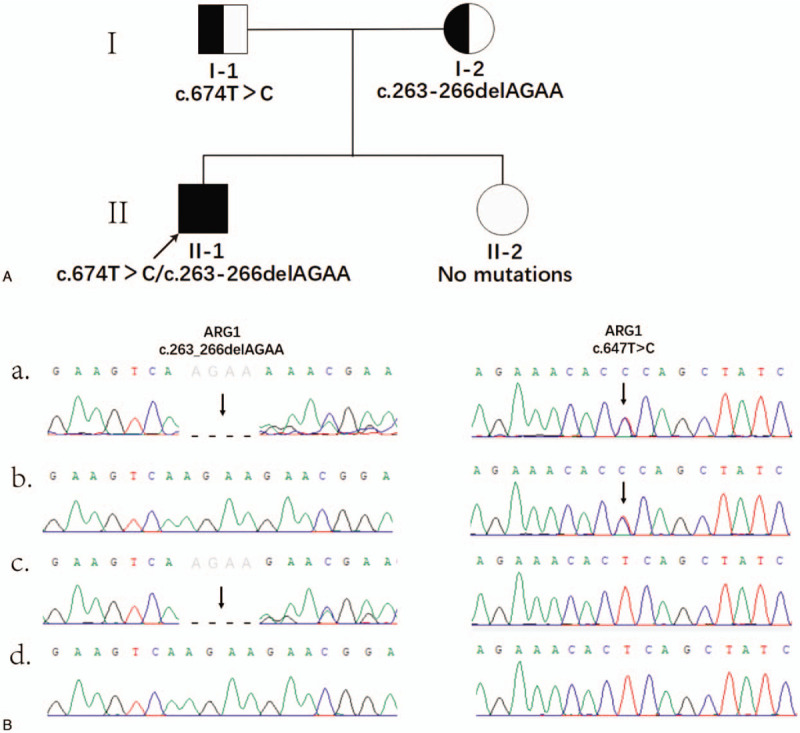
Identification of 2 compound heterozygous pathogenic variants within the ARG1 gene in this family. (A) Pedigree of this family. II-1 is the proband with 2 compound heterozygous mutations c.263-266delAGAA p.K88Rfs^∗^45 and c.674T>C,p.L216P in *ARG1* gene and his father(I-1) and mother (I-2) are definite carriers with variant c.674T>C,p.L216P and c.263-266delAGAA p.K88Rfs^∗^45, respectively. But his sister (II-2) has no mutations in the *ARG1* gene. (B) Sanger chromatograms (reverse sequence) showing the proband's variants c.263-266delAGAA p.K88Rfs^∗^45 and c.674T>C,p.L216P in *ARG1* gene (A), his mother's variant only in c.263-266delAGAA p.K88Rfs^∗^45 (B), his father's variant only in c.674T>C,p.L216P (C) and no variants in his sister's ARG1 gene.

So, he presented to neurology clinic of our hospital at the age of 13 years and 5 months. The physical examination revealed the sacral paraplegia, increased muscle tension in both lower limbs, muscle strength grade 5(Oxford Scale), and Knee reflex (+ +  +  + ) and ankle reflex (+ +  +  + ), ankle clonus, and Babinski sign (+) and Chaddock sign (+). However, the patient showed normal advanced neurological function, mutual aid movement, sensory system examination, urination, and defecation. A peripheral nerve examination revealed mildly reduced motor nerve conduction velocity (MCV) of bilateral tibial nerve (39.7 m/s left, 40.2 m/s right). We arranged the gene test for him, showing a novel compound heterozygous mutation in *ARG1* gene (c.263-266delAGAA, p.K88Rfs^∗^45;c.674T>C,p.L216P) (Fig. 1). The former has been reported to be pathogenic, whereas the latter is a newly developed pathogenic gene. And we simultaneously checked the blood and urine organic acid to find that blood arginine (183.70 μmol/L, normal range:1.00–70.00 μmol/L) and citrulline (53.04 μmol/L, normal range:5.50–45.00 μmol/L) increased and urine orotic acid (2.8 μmol/L, normal range:0.0–2.0 μmol/L) and uracil (10.5 μmol/L, normal range:0.0–8.0 μmol/L) also increased, but blood ammonia was normal (23 μmol/L, normal range: 9–33 μmol/L) (Table 1). To verify the family, we tested the *ARG1* gene for his parents to find out that his mother has a heterozygous mutation of c.263-266delAGAA but no c.674T > C mutation, whereas his father has a heterozygous mutation of c.674T > C but no c.263-266delAGAA mutation, which is a newly found gene mutation. However, the *ARG1* gene of his younger sister has no mutations (Fig. 1).

**Table 1:**
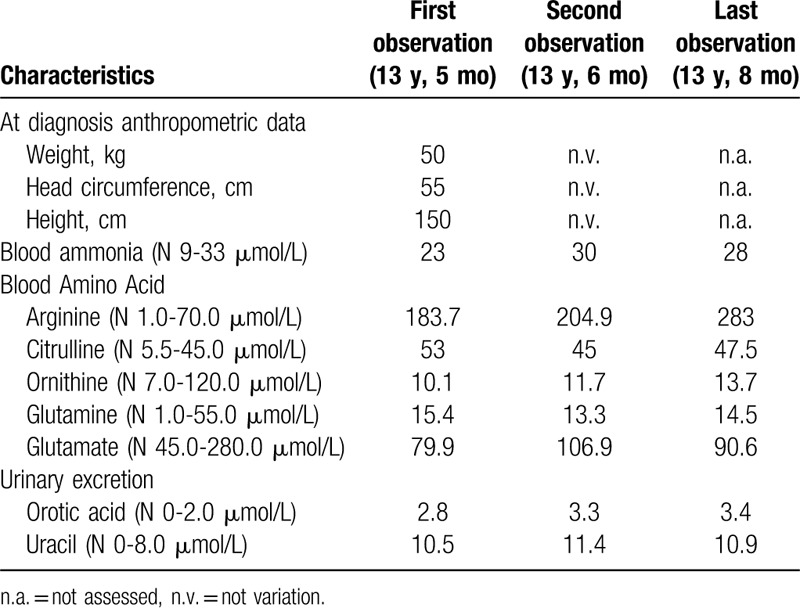
Growth index and longitudinal biochemical data.

Following the diagnosis, we recommended that the patient began a low-protein diet (0.8 g/kg/day). One month later, we reviewed the blood and urine organic acid and blood ammonia for the patient and the results showed blood arginine (204.9 μmol/L) and citrulline (45.0 μmol/L) were still high as well as the urine orotic acid (3.3 μmol/L) and uracil (11.4 μmol/L) (Table 1). As for drugs, we also recommended Baclofen single dose of 50 mg for 3 times per day and eperisone hydrochloride single dose of 50 mg for 3 times per day as well as botulinum toxin to relieve symptoms. However, although the related medicines and rehabilitation training were used for the patient for 3 months, his condition has not improved. Three months later, we detected the blood organic acid and blood ammonia for the patient again and the result showed blood arginine (283 μmol/L) and citrulline (47.5 μmol/L) were still high (Table 1).

## 3 Discussion

Agrininemia, or ARG1 deficiency, belongs to a rare autosomal recessive inherited urea cycle disorder, caused by a mutation in *ARG1* gene located on chromosome 6 (6q23), which was first reported by Terheggen et al.^[3]^ In our case, the gene test revealed that he carried a compound heterozygous mutation, c.(263-266delAGAA) (p.[K88Rfs^∗^45]) and c.(647T > C) (p.[L261P]) respectively from his parents. However, ARG1 of his younger sister has no mutations. Previously, the former was reported as a disease-causing mutation,^[4]^ leading to amino acid changing: p.K88Rfs^∗^45,which can cause protein disfunction, whereas the latter is a new variant, resulting in amino acid changing: p.L216P (Leucine>Proline). The effect of this novel mutation in vitro has not yet been investigated. By analysis of this gene mutation effects with the software MUTATION TASTER and POLYPHEN2, we found that it is highly conservative. We proved that this novel variant is pathogenic.

The clinical manifestations of arginase 1 deficiency are different from other urea cycle disorders. Children with argininemia can be normal in early childhood and gradually develop clinical symptoms later, including progressive spastic diplegia, intellectual impairment, growth retardation, and seizures et al.^[5,6]^ However, these manifestations are also obvious in other neurologic disorders like cerebral palsy, cerebellar ataxia, hereditary spastic paraplegia, and neurodegenerative diseases; therefore, it is easily misdiagnosed.^[7,8]^ Some patients show nonspecific symptoms in early childhood, such as protein intolerance, periodic vomiting, and retarded growth.^[9]^ In our case, the child did not have the above symptoms in his early childhood, making diagnosis even more difficult. However, he performed with the typical clinical features of progressive spastic paraparesis when he was 8 years’ old. He walked unsteadily and gradually progressed to a spastic diplegia. Currently, it is characterized by scissors gait. Cai et al^[10]^ reported a case of peripheral nerve damage, demonstrating it is one of the neurologic impairments of argininemia. In our patient, a peripheral nerve examination revealed mildly reduced MCV of bilateral tibial nerve. It also suggested that peripheral nerve was damaged in the patient.

The determination of blood organic acids by tandem mass spectrometry shows that hyperarginemia is the most critical diagnostic clue. It has been reported that blood arginine levels in some patients are 3 to 4 times higher than normal.^[11]^ We measured blood organic acids twice for the patient, all showing hyperarginemia, further supporting our diagnosis. Urinary orotic acid and uracil also elevate because of the stimulating effect of arginine on *N*-acetyglutamate synthesis.^[12]^ However, patients rarely show hyperammonemia. Some patients have experienced episodic hyperammonemia to variable degrees, which rarely leads to a life-threatening acute illness.^[5]^ The blood ammonia in our patient was normal in 2 consecutive tests, which is consistent with previous reports. However, we still need to monitor blood ammonia continually. Liver dysfunction is a common complication of arginemia, presenting elevated transaminases or bilirubin.^[11]^ The level of transaminases and bilirubin in our case is normal. But the coagulopathy is mild abnormal, including prolonged prothrombin time, increased international normalized ratio, and elevated d-dimer. There were not any signs of bleeding in our patient. However, it has been reported that some patients had slightly bleeding events like petechiae and ecchymosis.^[11]^ Until now, serious bleeding tendency has not been found in our patient. These atypical laboratory results are easily neglected. The reasons for coagulation disturbances in patients with agrininemia are not clear. Some studies reported that coagulation abnormalities were likely related to liver damage.^[13]^ Although major bleeding symptoms or apparent liver dysfunction are not apparently investigated in those patients with agrininemia, coagulation should also be detected.

Argrininemia is caused by a deficiency in arginase 1, which is a key enzyme of the final step in the urea cycle, catalyzing the hydrolysis of arginine to ornithine and urea. So far, the pathogenesis of neurological damage in patients with argininemia is unclear. Organs such as brain, liver, and kidney may be damaged by increased arginine and its metabolites in blood, including guanidino compounds and nitric oxide, which are known to be toxic.^[14]^ As the disease progresses, neurological damage becomes worse. Elevation of arginine increases the synthesis of nitric oxide, leading to oxidative damage, which can decrease brain metabolism by limiting Na + -K + adenosine triphosphatase. Moreover, guanidino compounds can affect neuronal survival and result in demyelination of corticospinal tract, which presents with upper motor neuron signs.^[6]^ In conclusion, the pathogenesis of neurological damage of our patient may be the cause of his all neurological presentations.

Argininemia is a potentially treatable disease of urea cycle disorders, especially in the early stage. Clinical treatments include arginine-restricted diet and related drugs including sodium benzoate and phenyl butyrate. After the clear diagnosis, we recommended the child with low-protein diet to decrease his blood arginine level. In addition, we suggested some symptomatic treatment including some drugs like Baclofen, eperisone hydrochloride and botulinum toxin as well as rehabilitation training. In a review, the treatment of spasticity with botulinum toxin injection was recommended.^[15]^ Kelle et al reported a 6-year-old girl with a diagnosis of argininemia and with a symptom of progressive spastic paraparesis. After 3 to 6 months of treatment with botulinum toxin injection, her spasticity improved.^[16]^ However, our patient muscle spasticity has no obvious improvement. We suspect that it is related to the short course of treatment.

After the limited protein intake for 3 months, we found that the level of arginine had not been reduced, and urinary orotic acid and uracil are still elevated. But the patient's condition did not become worse. We think that the patient did not have a strict low-protein diet, which was similar with a case reported by Jain-Ghai et al.^[17]^ Therefore, we still need to strongly recommend a low-protein diet and further observe the prognosis. Anyway, doctors need to pay more attention to ariginase 1 deficiency to achieve earlier diagnosis. Diagnostic basis mainly includes typical clinical manifestation and tandem mass spectrometry combined with molecular genetic testing.

## 4 Conclusions

We described a Chinese patient with argininemia who carried a novel mutation in *ARG1* gene. We also analyzed the effects of this novel mutation and proved it to be pathogenic. Argininemia is a potentially treatable disease of urea cycle disorders, especially in its early stages. Diagnostic basis mainly includes typical clinical manifestation and tandem mass spectrometry combined with molecular genetic testing. This report demonstrated that doctors should pay more attention to ariginase 1 deficiency to achieve earlier diagnosis.

## Acknowledgments

The authors thank the Natural Science Foundation of Shandong Province (ZR2016HM68) for the support.

## Author contributions

**Literature review:** Liang Jin, Liping Hu

**Writing – original draft:** Dongqing cui and Yanxia liu

**Writing – review & editing:** Lili Cao
